# A DNA2 mutation in the ATP-binding motif identified in a diagnostically unresolved individual

**DOI:** 10.3389/fmolb.2025.1706392

**Published:** 2025-11-21

**Authors:** Keisuke Saito, Yukiko Yatsuka, Ayuno Kawakami, Shinichiro Kumagaya, Nana Akiyama, Yasushi Okazaki, Kei Murayama, Hiroshi Ishikita

**Affiliations:** 1 Department of Applied Chemistry, The University of Tokyo, Tokyo, Japan; 2 Research Center for Advanced Science and Technology, The University of Tokyo, Tokyo, Japan; 3 Diagnostics and Therapeutics of Intractable Diseases, Intractable Disease Research Center, Graduate School of Medicine, Juntendo University, Tokyo, Japan; 4 Department of Metabolism, Chiba Children’s Hospital, Chiba, Japan; 5 Department of Pediatrics, Graduate School of Medicine, Juntendo University, Tokyo, Japan

**Keywords:** DNA2, TSC2, whole genome sequencing (WGS), medically unexplained symptoms (MUS), walker motif, P-loop

## Abstract

Many individuals with chronic, medically unexplained symptoms remain without a diagnosis, despite extensive clinical evaluations. Here, we present a framework integrating genome analysis with protein structural analysis to investigate such a case. Genome sequencing of a diagnostically unresolved individual identified a previously unreported DNA2 missense variant: T652R. This mutation lies within the Walker A motif (GxxxxGKT) of the helicase 1A domain, at an “x” position in the P-loop critical for ATP recognition. Structural analysis revealed that the introduced Arg652 sidechain displaces the conserved Lys654 from its canonical ATP-binding role and forms a new salt bridge with Asp973 in the helicase domain 2A. This interaction likely locks DNA2 in a closed conformation, impairing the dynamic domain movement essential for helicase activity. The case presented here demonstrates how structure-guided analysis of even a single missense variant can provide a basis of understanding the molecular origin of symptoms and help maximize the often underutilized diagnostic potential of genome sequencing.

## Introduction

Despite major advances in medical diagnostics, many individuals still suffer from persistent and often disabling health problems that remain unexplained. These patients with medically unexplained symptoms (MUS) find themselves outside the reach of conventional medicine (Smith and Dwamena, 2007; Husain and Chalder, 2021). They undergo repeated medical evaluations, hospital stays, and even international consultations, yet no clear diagnosis is ever provided to explain their symptoms. This kind of medical uncertainty has serious consequences—not only physical, but also psychological and social: patients may lose access to medical or social support, face disbelief fromhealthcare professionals, or even be accused of exaggerating or imagining their illness (Werner and Malterud, 2003).

A representative case involves an individual who, for more than 3 decades since early childhood, has experienced repeated episodes of severe cyclic vomiting, intense and chronic headaches, increased susceptibility to infections, chronic fatigue, and chronic unexplained pain all over the body. These symptoms have led to more than thirteen hospital admissions. The individual has consulted with a wide range of medical institutions in both domestic and international settings. Despite these efforts, no diagnosis or effective treatment has been identified. As a result, the individual remains medically classified as having no remarkable illness—substantially indistinguishable from a healthy person in medical records and histories—despite the undeniable presence of serious and persistent symptoms. Trapped in this diagnostic void, the person has faced worsening and unpredictable symptoms, while continuing to seek answers through any possible means.

This case reflects a broader challenge faced by many individuals suffering from chronic, unexplained symptoms. Even when such patients are fortunate enough to undergo modern genome sequencing, the diagnostic outcome often remains inconclusive. A large number of genetic variants identified through genome sequencing cannot yet be definitively linked to their physiological consequences. Moreover, even when a potentially relevant variant is identified, the molecular mechanisms it affects are often poorly understood—leaving patients without meaningful feedback or clarity, despite the considerable time, effort, and cost invested in testing (Amendola et al., 2020). On the other hand, integrating structural biology into variant interpretation offers a promising way forward, by linking genetic variants to their protein-level structural and functional consequences. In particular, structure-informed metrics based tools (e.g., AlphaMissense (Cheng et al., 2023) and AlphaFold 3 (Abramson et al., 2024)) can provide complementary evidence beyond conventional sequence-based *in silico* tools. These approaches have already proven valuable in clinically significant genes [e.g., *TP53* (Rotenberg et al., 2025) and *BRCA1* (Ramadane-Morchadi et al., 2025)], where they refine classification of variants of uncertain significance and enhance mechanistic interpretability. Importantly, such methods are increasingly viewed not merely as supportive but as an integral bridge between genotype and phenotype, paving the way for incorporation of structural evidence into standardized frameworks such as the American College of Medical Genetics (ACMG) and the Association for Molecular Pathology (AMP) guidelines (Varga et al., 2025).

Here we present a practical and widely applicable framework grounded in molecular biology. We begin with a comprehensive genome analysis of the diagnostically unresolved individual described above. Upon identification of a candidate mutation, we investigate its structural and functional consequences at the protein level. By linking genetic variation to molecular structural changes, this approach seeks to reveal potential disease mechanisms—supporting the fundamental right of individuals to understand their medical condition through the lens of protein structure and function.

## Results

### Genomic and transcriptomic analysis revealed two candidate variants

**FIGURE 1:**
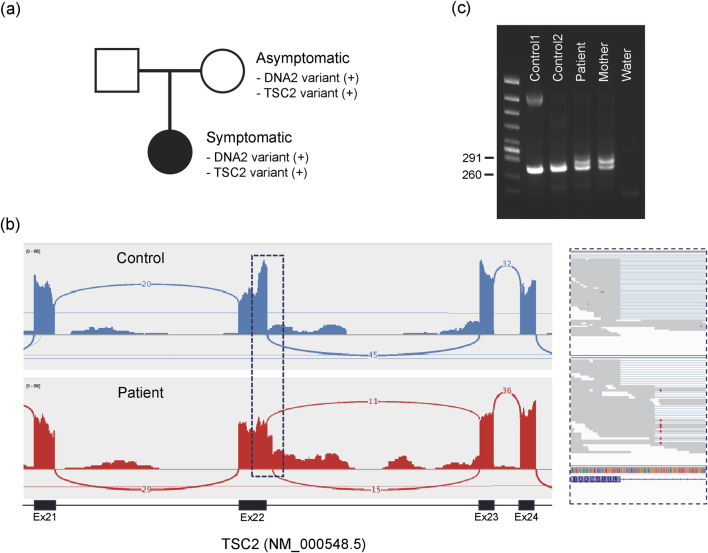
DNA2 and TSC2 variants identified in the symptomatic individual. **(a)** Both variants were maternally inherited. **(b)** RNA sequencing reveals splicing abnormalities at the end of exon 22 in TSC2 transcripts. The top panel shows a control sample; the bottom panel shows the patient. The boxed region is enlarged on the right. **(c)** Splicing abnormalities confirmed by RT-PCR. Canonical splicing between exons 22 and 23 yields a 248-bp fragment, whereas aberrant splicing with partial intron retention produces a 286-bp fragment, detected both in the patient and the mother.

To investigate the potential molecular origin of the patient’s unresolved symptoms, we conducted whole genome sequencing (WGS) and RNA sequencing using peripheral blood of the patient. The analysis revealed two heterozygous variants: one in the *DNA2* gene and another in the *TSC2* gene. Although these variants are not registered in ClinVar, both are present in gnomAD v4.1 at extremely low allele frequencies in the East Asian population (AF = 0.0000252 each). The variant identified in *DNA2* is a missense mutation at exon 13 (NM_001080449: c.1955C>G), resulting in a threonine-to-arginine substitution at position 652 of the protein (p.T652R). This variant maps to chromosome 10 at position 68431890 (GRCh38). To the best of our knowledge, this T652R variant has not been previously reported. This variant was confirmed as inherited from mother, who does not show any symptoms like the patient (Figure 1a). Meanwhile, the variant identified in *TSC2* is an intronic alteration (NM_000548.5: c.2545 + 42_2545 + 43delinsTT) on chromosome 16 (position 2074431–2074432, GRCh38). This variant was also confirmed to be maternally inherited (Figure 1a). Pathogenic variants in TSC2, which encodes the tumor suppressor protein tuberin, are known to cause tuberous sclerosis complex (TSC), an autosomal dominant disorder that affects multiple organs and exhibits a broad range of clinical presentations (Northrup et al., 2021). At least in our current analysis using blood, this variant appears to inhibit of canonical splicing of the TSC2 transcript (Figures 1b,c), however, its pathogenicity remains uncertain according to available clinical databases. In the patient, although hypomelanotic macules were observed during childhood and longitudinal depressions on the nails are present, typical symptoms such as cortical tubers, facial angiofibromas, epilepsy, or benign tumors in any organs have not been observed. Therefore, TSC2-related disease cannot be ruled out. Based on this context, we are proceeding with the analysis of DNA2 while concurrently investigating the TSC2 variant.

In conventional clinical practice, a DNA2 mutant such as T652R, having no previous reports linking it to a specific disease and lacking established functional assays, would typically be interpreted as a variant of uncertain significance. In the present study, however, we selected the T652R mutant DNA2 as the primary candidate for further investigation due to the functional relevance of DNA2 to mitochondrial DNA replication and repair, because *DNA2* encodes a nuclear-encoded helicase/nuclease essential for long-patch base excision repair and the maintenance of mitochondrial DNA integrity (Bae et al., 2001; Cejka et al., 2010; Hu et al., 2012). Below, we proceed with a detailed structural analysis of this mutant.

### Analysis of the arginine mutant protein conformations based on the mouse DNA2 crystal structure

Although the protein structure of human DNA2 has not yet been experimentally resolved, the crystal structure of mouse DNA2 provides a reliable model due to the high sequence conservation between the two species (Zhou et al., 2015). In particular, the region surrounding the threonine residue corresponding to human Thr652 (mouse Thr653) is highly conserved among mammals (Ronchi et al., 2013).

**FIGURE 2:**
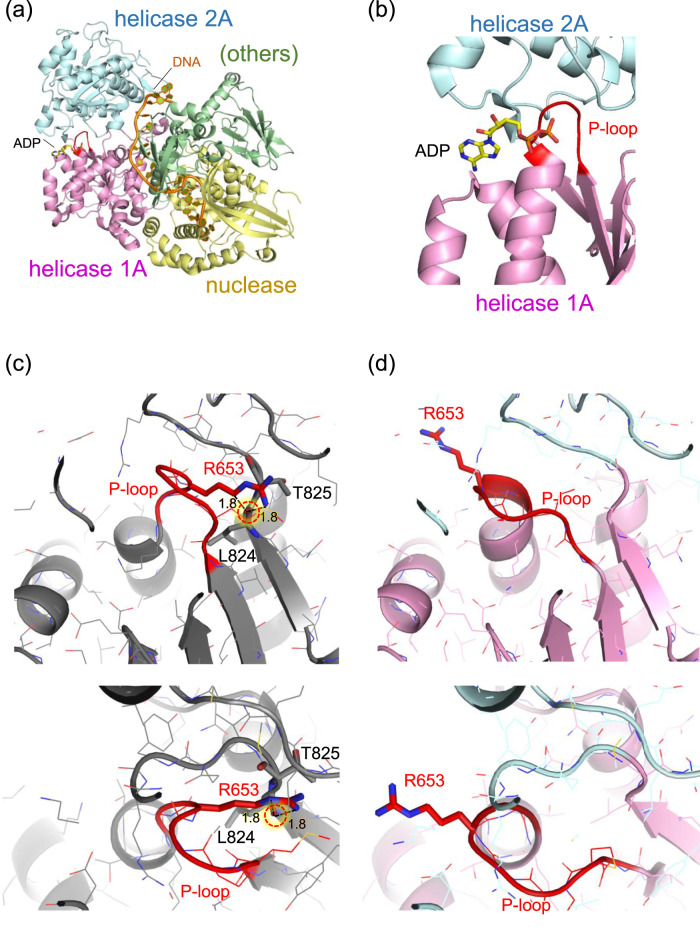
Structure of mouse DNA2 in complex. **(a)** Overall domain architecture of DNA2 with DNA and ADP (PDB: 5EAX). The helicase domain 1A (magenta), helicase domain 2A (cyan), and nuclease domain (gold) are shown along with bound DNA (orange) and ADP (CPK coloring). Other structural elements, including the oligonucleotide/oligosaccharide-binding domain, α1 domain, barrel domain, and stalk domain, are grouped as “others” (green). **(b)** Close-up view of the ADP-binding site adjacent to the P-loop (red) in the Walker A motif of helicase domain 1A. **(c)** Example of an Arg653 conformation while retaining the original (wild-type) P-loop conformation. The Arg653 side chain is positioned too close to the backbone carbonyl oxygen of Leu824 (1.8 Å), resulting in steric clashes. **(d)** Example of a plausible Arg653 conformation, in which the P-loop adopts a shifted conformation relative to the wild-type structure. The P-loop is shown in red. Major steric clashes are indicated by red dotted circles with labeled distances (Å).

In the mouse DNA2 structure, Thr653 is located in a short loop (Met650–Thr653) that connects a β-strand (Tyr644–Gly649) to an α-helix (Gly654–Cys669) within the helicase 1A domain. Remarkably, the amino acid sequence in this region, Met650–Pro651–Gly652–Thr653–Gly654–Lys655, matches the consensus GxxxxGKT motif characteristic of the “Walker A motif”, a common structural signature in ATP-binding and hydrolysis sites, where the terminal threonine (T) is sometimes replaced with serine (S) (Walker et al., 1982). In this context, the short loop containing Thr653 corresponds to the phosphate-binding loop, “P-loop,” of the Walker A motif (Saraste et al., 1990). This P-loop forms a sharp ∼180° turn, allowing the strand and helix to run nearly in parallel (Figure 2). The sidechain of Thr653 is oriented toward the inner face of the loop, compactly accommodated in a narrow space framed by the backbone groups. The small and polar nature of the threonine sidechain enables this fit without inducing steric strain.

In contrast, substitution of Thr653 with the bulkier, positively charged arginine introduces significant steric hindrance. The guanidinium group of arginine cannot be accommodated within the original loop pocket. Rotamer analysis using Maestro (Maestro, 2024) generated multiple conformations of the arginine sidechain, but none showed favorable stability scores. Even the most energetically favorable conformation appeared structurally incompatible with the surrounding loop geometry (Figure 2c). These rotamers, in which the arginine sidechain points away from the ATP/ADP-binding site, were also considered. However, this orientation causes steric clashes with adjacent β-strand segments (Val821–Leu824) near Thr825. Thus, in either rotameric state, the arginine sidechain imposes spatial constraints that are incompatible with the native fold.

The most plausible structure of the arginine conformation was obtained using AlphaFold 3 (Abramson et al., 2024), which shows that, in the absence of ATP/ADP, the only seemingly reasonable orientation of the arginine sidechain extends toward the adjacent ATP/ADP binding pocket, resulting in a notable displacement of the backbone groups in the P-loop (Figure 2d).

While these observations for T653R mutation are based on the mouse DNA2 structure, these observations suggest that the T652R mutation in human DNA2 similarly leads to structural perturbation that likely affects ATP/ADP binding by inducing local conformational changes in a highly conserved functional domain. With the likely conformation of the introduced arginine now established, further structural analysis, specifically of the ATP/ADP-binding site, is presented below, based on the currently most plausible structure of T652R mutant human DNA2, which was generated using AlphaFold 3 (Abramson et al., 2024) and refined using the CHARMM force field (Brooks et al., 1983).

### Implication from the ATP/ADP binding geometry in human T652R DNA2

**FIGURE 3:**
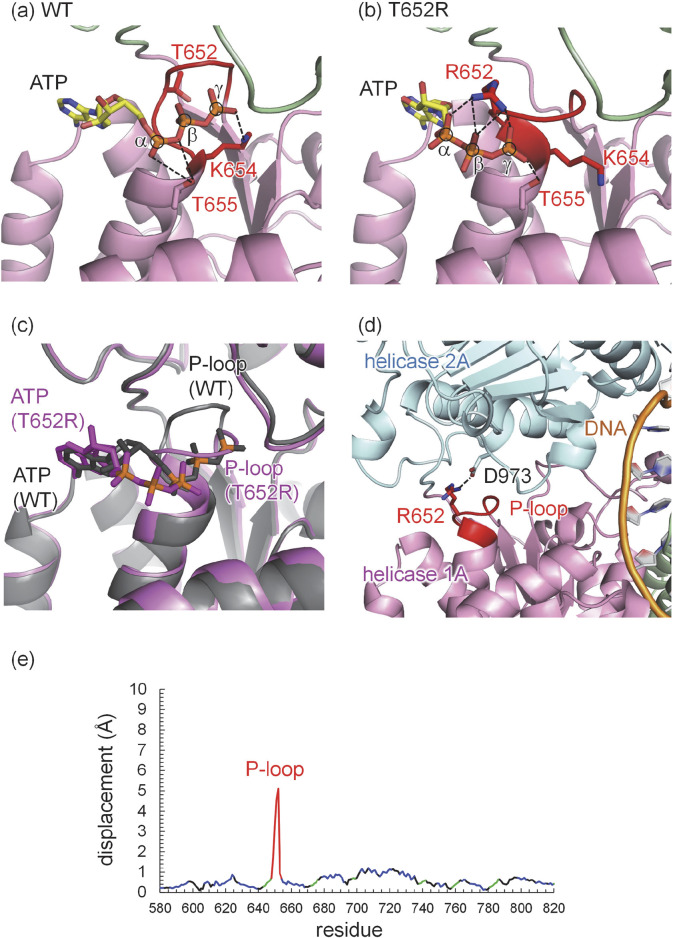
Refined structure of human DNA2 with ATP. **(a)** Wild type (WT). **(b)** T652R mutant. **(c)** Superimposed structures of the wild type (gray) and the T652R mutant (magenta). Phosphate atoms of ATP, α, β, and γ, are shown in orange and circled. Dotted lines indicate electrostatic interactions, including H-bonds and salt bridges. **(d)** Salt bridge between Arg652 in the helicase 1A domain and Asp973 in the helicase 2A domain revealed in the T652R mutant structure of human DNA2. The dotted line indicates the salt bridge between the Arg652 side chain (red) and the Asp973 side chain. Domains are colored as follows: helicase 1A (magenta), helicase 2A (cyan), DNA (orange), and the P-loop is highlighted in red. **(e)** Displacements of Cα atoms between the wild-type and T652R human DNA2 structures: P-loop (red), helix (blue), β-sheet (green), and others (black).

Human and mouse DNA2 exhibit high overall homology (Supplementary Figure S1). In particular, the ATP-binding active site is highly conserved, and the ATP binding site of the wild-type human DNA2 structure closely superimposed on that of the mouse DNA2 structure (Supplementary Figure S2). In the wild-type human DNA2 structure, ATP binds near the P-loop. Upon the T652R mutation, structural changes are observed predominantly in the P-loop (Figure 3e). Specifically, the conserved lysine residue, Lys654, in the consensus GxxxxGKT motif, forms a salt bridge with the oxygen of the γ-phosphate—the terminal phosphate of ATP, as commonly observed in canonical Walker A motifs (Walker et al., 1982; Saraste et al., 1990) (Figure 3a).

In the T652R mutant structure, the Arg652 sidechain partially occupies the region where ATP binds in the wild-type structure. In this mutant, ATP interacts predominantly with the Arg652 sidechain rather than with the conserved lysine, forming three salt bridges with all three phosphate groups of ATP (Figure 3b). Notably, the presence of positively charged Arg652 at the ATP-binding site displaces positively charged Lys654, normally responsible for γ-phosphate recognition, away from the ATP molecule, as the negative charges of the phosphate groups are already compensated by Arg652.

Lys654 plays a critical role in DNA2 activity. Mutational studies have shown that substitution of Lys654 with alanine (K654A) or arginine (K654R) abolishes ATPase and helicase activity (Bae et al., 1998), and the K654E mutant eliminates enzymatic activity entirely (Budd et al., 1995). Moreover, cells expressing the K654E mutant of DNA2 could not survive (Bae et al., 2002). These findings suggest the essential role of Lys654 in ATP binding and hydrolysis. Although Arg652 in the T652R mutant partially substitutes for the role of Lys654 in ATP binding, the absence of Lys654 from its canonical position leads to a clearly altered ATP-binding mode. This altered configuration is likely to affect not only ATP affinity but also the coupling between ATP hydrolysis and mechanical translocation.

Despite these changes, the ATP head group in the mutant exhibits only minor displacement (∼1 Å) toward the solvent relative to the wild-type structure. In contrast, the location of the phosphate groups, specifically the terminal γ-phosphate moves ∼6 Å away toward the bulk from its original position in the wild type structure (Figure 3c). Indeed, the position of the γ-phosphate in the T652R structure nearly corresponds to the position of the α-phosphate in the wild type structure (Figures 3a,b). Consequently, this alters the interaction of the highly conserved threonine, which is adjacent to the lysine of the consensus GxxxxGKT motif (Saraste et al., 1990), i.e., Thr655. In the wild type human DNA2 structure, Thr655 forms an H-bond with the oxygen between α-phosphate and β-phosphate (Figure 3a), whereas it forms an H-bond with one of the oxygen atoms of the γ-phosphate (Figure 3b).

To evaluate whether the introduction of a positively charged residue at position 652 alters the protonation pattern, the protonation states of all titratable residues are analyzed in both wild-type and T652R mutant structures. All residues retain their standard protonation states—i.e., acidic residues deprotonated and basic residues protonated—except for Glu298, which is protonated due to its proximity to the phosphate backbone of bound DNA in both wild-type and T652R mutant structures. However, as Glu298 is located ∼50 Å from the ATP-binding site and no changes in the protonation pattern is observed upon mutation, the structural changes caused by the T652R mutation can be attributed specifically to local rearrangements at the ATP/ADP-binding site. These observation suggest that, while both the wild-type and T652R mutant structures support ATP binding near the P-loop, the binding modes differ substantially: Lys654 is the principal interacting residue in the wild type, whereas Arg652 plays the dominant role in the mutant.

## Discussion

### Impact of the T652R mutation on helicase domain dynamics

**FIGURE 4:**
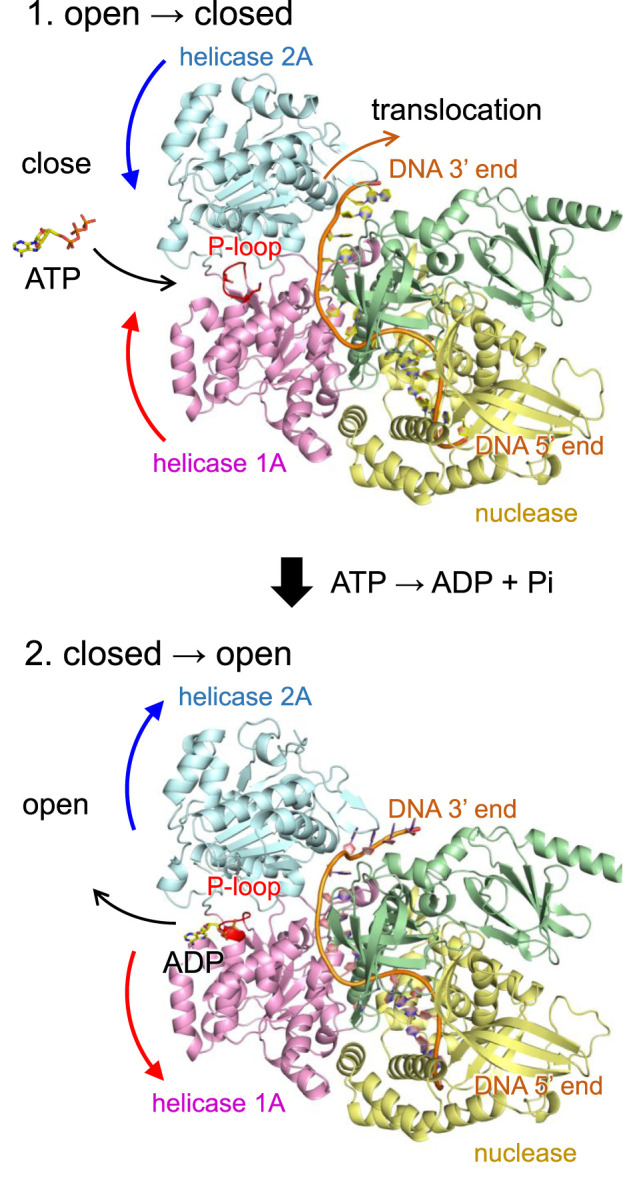
Mechanism of DNA translocation in DNA2 based on the wild-type human DNA2 structure. (Top) ATP binding induces a conformational change in DNA2, bringing the helicase 1A and 2A domains into a closed conformation. In this state, ATP is hydrolyzed to ADP and inorganic phosphate (Pi), and the released energy drives the translocation of DNA by one nucleotide in the 5′to 3′direction. (Bottom) Following translocation, DNA2 reverts to the open conformation, facilitating the release of ADP and resetting the system for the next cycle. Curved arrows indicate orientations of molecular movements. In the T652R mutant, this conformational reopening is suppressed due to the formation of a salt bridge between Arg652 and Asp973.

The helicase activity of DNA2 is driven by a series of dynamic conformational changes involving the helicase 1A and 2A domains (Zhou et al., 2015). Upon ATP binding, these two domains undergo a domain closure movement that secures the bound single-stranded DNA (ssDNA) between them. Subsequent ATP hydrolysis and phosphate release trigger domain reopening, which enables the enzyme to advance by one nucleotide along the DNA strand (Figure 4). This cyclical opening and closing motion, often referred to as a “power stroke”, underlies the processive translocation and unwinding of ssDNA by DNA2.

Remarkably, in the T652R mutant, structural analysis reveals that the introduced arginine sidechain at position 652 (located in the P-loop of helicase domain 1A) forms a salt bridge with Asp973 in the helicase 2A domain (Figures 3d and Supplementary Figure S3). As salt bridges are known to stabilize interdomain or subunit interactions in proteins, this interaction likely artificially stabilizes the closed conformation of DNA2, effectively “locking” the two domains together. As a result, the essential reopening step following ATP hydrolysis is likely impaired or inhibited. Although ATP binding may still occur in the T652R mutant, the coupling between ATP hydrolysis and mechanical motion is uncoupled, leading to a functional loss of helicase activity.

While DNA2 plays roles in both nuclear and mitochondrial DNA metabolism, its helicase activity is particularly essential in mitochondria, where alternative repair mechanisms are limited (Zheng et al., 2008). Mitochondrial DNA (mtDNA), unlike nuclear DNA, is continually exposed to oxidative damage and structural stress during replication (Alexeyev et al., 2013), and DNA2 is required for processing flap structures and resolving replication intermediates. A defect in DNA2-mediated helicase function may compromise the repair and maintenance of mtDNA, leading to progressive mitochondrial dysfunction. This could result in multi-system symptoms of unclear origin, as mitochondrial impairment can affect a broad range of cellular functions. Thus, the structural mechanism identified here may explain how a single amino acid substitution in DNA2 can lead to complex, diagnostically elusive phenotypes.

Both the mouse DNA2 crystal structure and the modeled human DNA2 structure correspond to the closed conformation, in which ATP/ADP is bound. To our knowledge, an open conformation of DNA2 has not yet been reported. This is likely because the open state, which becomes more flexible and disordered upon loss of nucleotide binding, is intrinsically more difficult to capture at high resolution. In contrast, the closed conformation is stabilized by nucleotide binding and therefore more structurally ordered. Because no open conformation structure is available, a direct quantitative comparison of the structural stability between the open and closed conformations cannot be meaningfully performed. Nevertheless, stability differences between the wild-type and T652R mutant proteins can be inferred from their intramolecular interactions. In general, the H-bond energy is ∼1 kcal/mol for interactions between uncharged polar groups, ∼2 kcal/mol for interactions between charged and uncharged polar groups, and ∼4 kcal/mol for interactions between two charged groups (Perrin and Nielson, 1997). In the T652R mutant, the introduced Arg652 in helicase domain 1A forms a salt bridge with Asp973 in helicase domain 2A, an interaction that is absent in the closed-state wild-type protein. This additional charged interaction is expected to increase the stability of the closed conformation relative to that of the wild type.

### Comparison with other mutants near the P-loop

The present study demonstrates that the T652R mutation in DNA2, despite being located within the conserved GxxxxGKT sequence of the Walker A motif, does not completely abolish ATP binding. Although the P-loop is deformed and the binding mode is altered, such that the introduced Arg652 sidechain forms multiple salt bridges with all three phosphate groups of ATP, ATP can still be accommodated. This partial preservation of binding is likely due to the mutation occurring at a flexible “x” position within the motif, where some structural tolerance remains.

In contrast, a more severe phenotype has been reported for a nearby mutation, T655A, located just three residues downstream (Tarnauskaitė et al., 2019). Thr655 lies immediately after the conserved lysine in the GxxxxGKT motif and plays a direct role in coordinating the γ-phosphate of ATP through H-bond. Substitution of this highly conserved threonine with alanine eliminates this H-bond interaction, leading to a substantial loss of ATP binding and, ultimately, helicase activity.

Clinically, the T655A mutation leads to a severe mitochondrial disorder. The patient exhibited early-onset progressive microcephaly, developmental delay, growth retardation, and systemic mitochondrial dysfunction (Tarnauskaitė et al., 2019). Functional assays revealed dramatic reductions in DNA2 ATPase and helicase activity, alongside defects in mitochondrial DNA replication and maintenance. These findings directly link the structural and biochemical role of Thr655 to the observed disease pathology.

The comparison between T652R and T655A is instructive. Although both mutations occur within the same structural motif and in close sequence proximity, their functional consequences differ markedly. This distinction is not readily apparent from sequence analysis alone. However, once visualized in the context of the three-dimensional protein structure, the reasons become clear: Thr655 directly coordinates ATP via H-bond, while Thr652, though nearby, occupies a more permissive position within the motif and allows for a partially retained, though altered, binding interaction.

To the best of our knowledge, aside from the T652R variant described here and the previously reported T655A substitution (Tarnauskaitė et al., 2019), no other missense variants have been identified that target the ATP-binding pocket of human DNA2.

### Lines of evidence considered in variant assessment

In interpreting the potential impact of the DNA2 T652R variant, we systematically evaluated the major categories of evidence recommended in variant-interpretation frameworks.

i. Clinical presentation: The most direct evidence comes from the individual carrying the T652R substitution, who has suffered from severe, recurrent, and chronic symptoms, including cyclic vomiting, persistent headaches, heightened susceptibility to infection, chronic fatigue, and diffuse unexplained pain, for more than 3 decades since early childhood. This consistent and long-term phenotype suggests an underlying genetic cause.
ii. Genomic evidence: Whole-genome sequencing revealed a heterozygous missense variant, T652R, in DNA2. This variant is rare in population databases and has not been previously reported in association with any human disease. Owing to this absence of prior knowledge, conventional variant-classification pipelines categorize it as a variant of uncertain significance.
iii. Functional evidence: No biochemical or cell-based functional assays for this specific mutation have been reported. These lines of evidence therefore cannot currently be applied.
iv. Structural evidence: To assess the potential molecular consequences of the T652R substitution, we constructed a structural model of human DNA2 based on AlphaFold 3 (Abramson et al., 2024) and compared it with the available mouse DNA2 crystal structure. The analysis revealed that the substitution perturbs the canonical ATP-binding geometry, displaces the highly conserved Lys654, and creates a non-native Arg652…Asp973 salt bridge. These alterations are likely to stabilize an inactive, closed conformation of the protein and thereby impair helicase function.

Taken together, the integration of clinical phenotype, genomic context, and structural analysis provides complementary and mutually reinforcing evidence supporting the functional impact of the T652R variant and its likely contribution to the patient’s complex disease phenotype.

### Limitations in this study

This DNA2 variant was inherited from the asymptomatic mother and present in gnomAD v4.1 at extremely low allele frequencies. These data demonstrate the presence of asymptomatic individuals carrying the variant. Differences in clinical presentation among individuals harboring the same variant have been reported not only in DNA2 but also across various other genes. For example, regarding DNA2, the clinical manifestations of a brother and sister were described and slightly different (Ronchi et al., 2013). We consider that, in addition to genetic factors, environmental influences may contribute to phenotypic variability. Furthermore, it is important to consider the possibility that the currently asymptomatic mother may develop symptoms in the future.

## Conclusions

The present study demonstrates a practical framework that integrates genome analysis with structural analysis of the encoded protein to elucidate the molecular basis of disease in a diagnostically unresolved individual who had experienced chronic, medically unexplained symptoms for over 3 decades. After extensive clinical investigations across multiple institutions, genome sequencing was finally performed, revealing a previously unreported missense variant in the *DNA2* gene, T652R.

This mutation occurs at one of the “x” positions within the highly conserved Walker A motif, GxxxxGKT, located in helicase 1A domain of DNA2. Structural analysis revealed that the T652R substitution induces significant conformational changes in the P-loop region (the “xxxx” segment), which is essential for ATP recognition. In the wild-type protein, the conserved Lys654 (the “K” in the motif) coordinates the γ-phosphate of ATP through a salt bridge. In the T652R mutant, however, the introduced Arg652 replaces Lys654 as the primary residue for phosphate binding, altering the canonical ATP-binding geometry. Remarkably, Arg652 in helicase domain 1A also forms a salt bridge with Asp973 in helicase domain 2A, likely stabilizing the closed conformation of DNA2 and preventing the domain opening required for helicase activity.

Despite the widespread availability of genome sequencing, the functional interpretation of identified variants remains a major bottleneck in personalized medicine. The approach presented here demonstrates that structure-guided analyses can offer diagnostically unresolved patients a scientifically grounded explanation of their symptoms, in line with recent perspectives highlighting the role of structural biology in improving variant assessment and clinical interpretation (Varga et al., 2025). This understanding is not only of value to patients, who have a right to understand the molecular basis of their condition, but also to physicians, who are not always experts in protein biochemistry. Integrating such molecular-level analyses into diagnostic workflows may enhance individualized patient care and promote a more collaborative understanding between patients, physicians, and researchers across disciplines.

## Methods

### Ethics approval and consent to participate

The study was conducted under ethical agreement and permission of the review board in the involved facilities (Juntendo University, Chiba Children’s Hospital, and Saitama Medical University). All procedures were conducted following the relevant rules and guidelines and in accordance with the Declaration of Helsinki. Written informed consent was obtained from the patient.

### Genomic and transcriptomic analysis

WGS was performed using genomic DNA derived from the patient and analyzed as previously described (Ozaki et al., 2024). Transcriptome analysis was also performed using blood RNA extracted by PAXgene Blood RNA System (QIAGEN) from the patient. After quality check by TapeStation 4,200 (Agilent) and Qubit 4.0 (Thermo Fisher Scientific), cDNA library was generated using Illumina Stranded Total RNA Prep Ligation with Ribo-Zero Plus (Illumina). Sequencing was performed using 150 bp paired-end reads on a NovaSeq6000 (Illumina). Data analysis was performed as previously described (Ozaki et al., 2024). In addition, splicing aberrations were investigated using FRASER (Mertes et al., 2021).

### Confirmation of segregation

Sanger sequencing was performed using genomic DNA derived from the patient and the parents to confirm of segregation in the variants which detected in the patient. Primer sequences are shown below. DNA2-F:5′-CATCTTTAAAGGGAAAGGAAAACA-3′, DNA2-R:5′-GACCAAGACTCTTTGGAAATGG-3′, TSC2-F:5′-ATGGTCTACTGCCTGGAGCA-3′, TSC2-R:5′-AGTCACAGTCTGGGGGAAGC-3’.

### Reverse transcription PCR (RT-PCR)

Reverse transcription PCR (RT-PCR) was performed to validate the aberrant splicing event identified by RNA sequencing. First, cDNA was synthesized using ReverTra Ace qPCR RT Master Mix with gDNA Remover (FSQ-301, TOYOBO), and then PCR amplification was carried out with KOD-Plus- DNA polymerase (KOD-201, TOYOBO). Primer sequences are listed below: TSC2-RTPCR-F: 5′-ATGGTCTACTGCCTGGAGCA-3′, TSC2-RTPCR-R: 5′-GCGAACACACTGGCATACTG-3’. The sequences of the PCR-amplified fragments were confirmed by direct sequencing using the same primers.

### Structural analysis of DNA2 proteins

The mouse T653R mutant structure in the absence of ATP was generated using AlphaFold 3 (Abramson et al., 2024), resulting in the arginine side chain being oriented toward the ATP-binding site. Atomic coordinates of the Fe_4_S_4_ cluster were incorporated into the resulting structures from the mouse DNA2 crystal structure (PDB code, 5EAX) (Zhou et al., 2015). The same arginine conformation was adopted in the initial structure of the human T652R mutant, also generated using AlphaFold 3 (Abramson et al., 2024). Human DNA2 structures for both the wild-type and T652R mutant in the presence of ATP were generated using AlphaFold 3 (Abramson et al., 2024). Hydrogen atoms were added and energetically optimized using CHARMM (Brooks et al., 1983). Atomic partial charges of amino acids and ATP were adopted from the all-atom CHARMM22 parameter set (Brooks et al., 1983). Atomic charges for the Fe_4_S_4_ cluster were adopted from previous studies (Kawashima and Ishikita, 2018). In human DNA2, the geometries of ATP and the Thr644–Cys659 region, including the P-loop, were energetically optimized. The resulting atomic coordinates of these DNA2 structures are provided in the Supplementary Material.

### Protonation pattern

The protonation states of titratable residues were calculated by solving the linear Poisson-Boltzmann equation using the MEAD program (Bashford and Karplus, 1990) using a three-step grid-focusing procedure with resolutions of 2.5 Å, 1.0 Å, and 0.3 Å. To maintain consistency with previous computational results (e.g., (Kawashima and Ishikita, 2018; Nishikawa et al., 2023)), all calculations were performed at 300 K, pH 7.0, and an ionic strength of 100 mM. The dielectric constants used were four for the protein interior and 80 for water. The p*K*_a_ values of titratable sites in the protein were determined by adding the calculated p*K*_a_ shifts relative to a reference system to known reference p*K*_a_ values: 12.0 for Arg, 4.0 for Asp, 9.5 for Cys, 4.4 for Glu, 10.4 for Lys, 9.6 for Tyr (Nozaki and Tanford, 1967), and 7.0 and 6.6 for the N_ε_ and N_δ_ atoms of His, respectively (Tanokura, 1983a; Tanokura, 1983b; Tanokura, 1983c).

The equilibrium protonation probability of a titratable group *i* is defined by the thermodynamic average over all 2^*n*^ protonation microstate (where *n* is the total number of titratable sites in the protein), as given in Equation 1:

$$\langle x_{i}\rangle =\frac{\sum_{\vec{q}}x_{i}\exp\left[-\beta \sum_{\mu }\left(x_{\mu }\Delta G_{\text{intr}/\mu }+\frac{1}{2}\sum_{\nu \neq \mu }q_{\mu }q_{\nu }W_{\mu \nu }\right)\right]}{\sum_{\vec{q}}\exp\left[-\beta \sum_{\mu }\left(x_{\mu }\Delta G_{\text{intr}/\mu }+\frac{1}{2}\sum_{\nu \neq \mu }q_{\mu }q_{\nu }W_{\mu \nu }\right)\right]}$$ (1)

Here, *β* = (*k*_B_ *T*)^−1^ is the inverse thermal energy, *k*_B_ is the Boltzmann constant, and *T* is the temperature. *x*_*i*_ is the protonation state of site *i* (1 if protonated, 0 if deprotonated), Δ*G*_intr/*μ*_ and Δ*G*_intr/*ν*_ are the intrinsic protonation free energies of groups *μ* and *ν*, respectively, relative to reference states, *q*_*μ*_ and *q*_*ν*_ represent the charges associated with the protonation states of sites *μ* and *ν*, respectively, and *W*_*μν*_ is the pairwise electrostatic interaction energy between sites *μ* and *ν*. The summation over *ν* ≠ *μ* accounts for the interactions of site *μ* with all other titratable sites *ν* in the protein.

These electrostatic free energy terms are obtained by solving the linear Poisson-Boltzmann equation for the protein electrostatic environment. Because the number of microstates grows exponentially with *n*, direct evaluation of Equation 1 by enumerating all possible states is not practical in realistic applications. Therefore, the thermodynamic average in Equation 1 was evaluated by Monte Carlo sampling implemented in Karlsberg (Rabenstein et al., 1998). This procedure yields the Boltzmann-weighted equilibrium ensemble of protonation states directly, without the need for any explicit time-dependent equilibration procedure. As a result, the protonation probabilities reported in the present study inherently represent the thermodynamic equilibrium distribution of states.

## Data Availability

The raw data supporting the conclusions of this article will be made available by the authors, without undue reservation.
