# Supplementary figures and images for: A DNA2 mutation in the ATP-binding motif identified in a diagnostically unresolved individual
Source: Front Mol Biosci. 2025 Nov 21;12:1706392. doi: 10.3389/fmolb.2025.1706392 (PMC12678126; doi:10.3389/fmolb.2025.1706392)

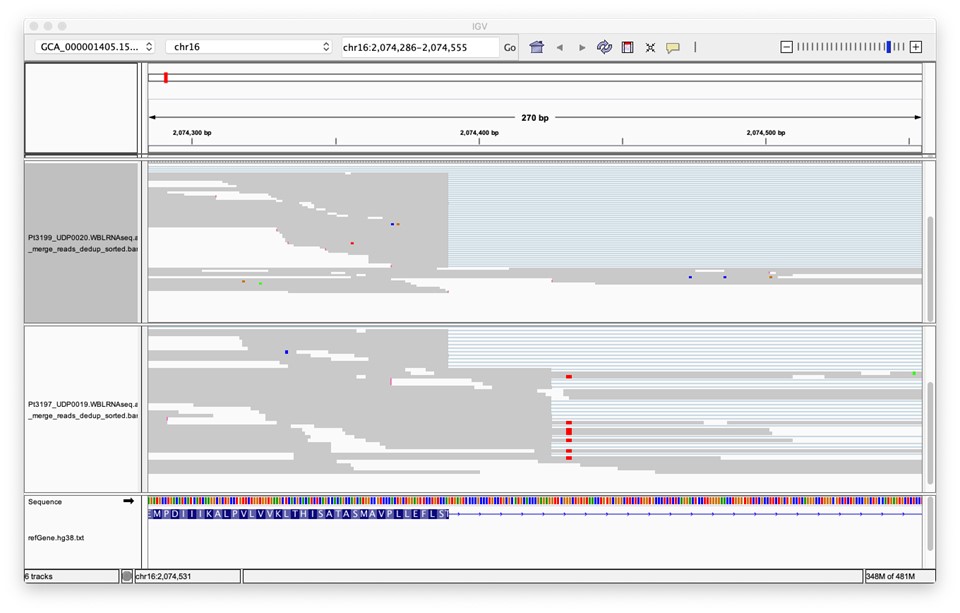

Supplement: Supplementary file 1 [file Image3.jpeg]

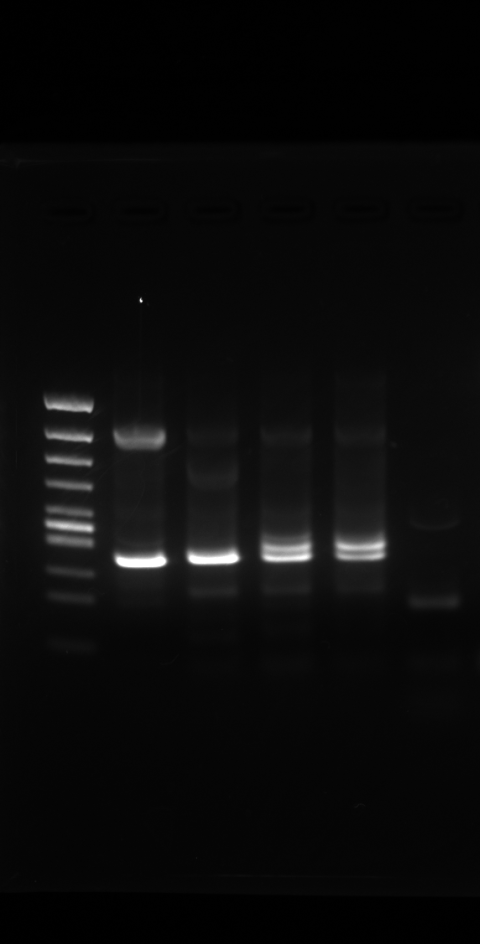

Supplement: Supplementary file 2 [file Image4.tif]

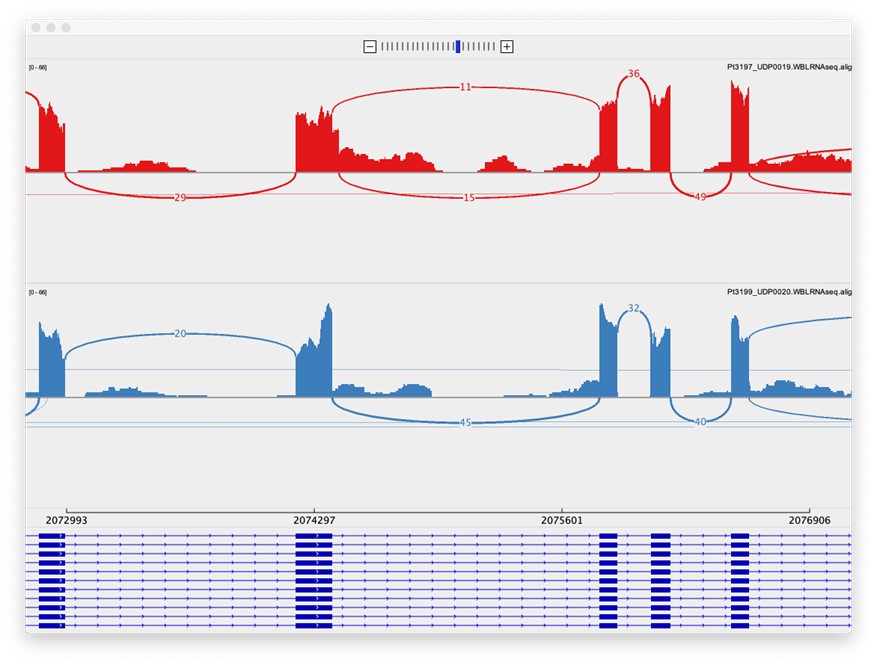

Supplement: Supplementary file 4 [file Image2.jpeg]
