# Supplementary material for: A DNA2 mutation in the ATP-binding motif identified in a diagnostically unresolved individual
Source: Front Mol Biosci. 2025 Nov 21;12:1706392. doi: 10.3389/fmolb.2025.1706392 (PMC12678126; doi:10.3389/fmolb.2025.1706392)
Supplement: Supplementary file 5 [file Image1.pdf]

```
DNA2_MOUSE      MEPLDELDLLLLEEDGGAEAVPRVELLRKKADALFPETVLSRGVDNRYLVLAVETSQNER 60
DNA2_HUMAN      MEQLNELELLMEKSFWEEAELP-AELFQKKVVASFPRTVLSTGMDNRYLVLAVNTVQNKE 59
                ** *:**:**: :.      :* .**::**. * **.**** *:*********:* **:.

DNA2_MOUSE      GAEEKRLHVTASQDREHEVLCILRNGWSSVPVEPGDIVHLEGDCTSEPWIIDDDFGYFIL 120
DNA2_HUMAN      GNCEKRLVITASQSLENKELCILRNDWCSVPVEPGDIIHLEGDCTSDTWIIDKDFGYLIL 119
                *  **** :****. *:: ******.*.*********:********:.****.****:**

DNA2_MOUSE      YPDMMISGTSVASSIRCLRRAVLSETFRGSDPATRQMLIGTILHEVFQKAISESFAPERL 180
DNA2_HUMAN      YPDMLISGTSIASSIRCMRRAVLSETFRSSDPATRQMLIGTVLHEVFQKAINNSFAPEKL 179
                ****:*****:******:**********.************:*********.:*****:*

DNA2_MOUSE      QELALQTLREVRHLKEMYRLNLSQDEILCEVEEYLPSFSKWAEDFMRKGPSSEFPQMQLS 240
DNA2_HUMAN      QELAFQTIQEIRHLKEMYRLNLSQDEIKQEVEDYLPSFCKWAGDFMHKNTSTDFPQMQLS 239
                ****:**::*:****************  ***:*****.*** ***:*..*::*******

DNA2_MOUSE      LPSDGSNRSSPCNIEVVKSLDIEESIWSPRFGLKGKIDVTVGVKIHRDCKMKYKVMPLEL 300
DNA2_HUMAN      LPSDNSKDNSTCNIEVVKPMDIEESIWSPRFGLKGKIDVTVGVKIHRGYKTKYKIMPLEL 299
                ****.*: .*.*******.:***************************. * ***:*****

DNA2_MOUSE      KTGKESNSIEHRSQVVLYTLLSQERREDPEAGWLLYLKTGQMYPVPANHLDKRELLKLRN 360
DNA2_HUMAN      KTGKESNSIEHRSQVVLYTLLSQERRADPEAGLLLYLKTGQMYPVPANHLDKRELLKLRN 359
                ************************** ***** ***************************

DNA2_MOUSE      WLAASLLHRVSRAAPGEEARLSALPQIIEEEKTCKYCSQIGNCALYSRAVEEQGDDASIP 420
DNA2_HUMAN      QMAFSLFHRISKSATRQKTQLASLPQIIEEEKTCKYCSQIGNCALYSRAVEQQMDCSSVP 419
                 :* **:**:*::*. ::::*::****************************:* * :*:*

DNA2_MOUSE      EAMLSKIQEETRHLQLAHLKYFSLWCLMLTLESQSKDNRKTHQSIWLTPASELEESGNCV 480
DNA2_HUMAN      IVMLPKIEEETQHLKQTHLEYFSLWCLMLTLESQSKDNKKNHQNIWLMPASEMEKSGSCI 479
                 .**.**:***:**: :**:******************:*.**.*** ****:*:**.*:

DNA2_MOUSE      GNLVRTEPVSRVCDGQYLHNFQRKNGPMPATNLMAGDRIILSGEERKLFALSKGYVKKMN 540
DNA2_HUMAN      GNLIRMEHVKIVCDGQYLHNFQCKHGAIPVTNLMAGDRVIVSGEERSLFALSRGYVKEIN 539
                ***:* * *. *********** *:*.:*.********:*:*****.*****:****::*

DNA2_MOUSE      KAAVTCLLDRNLSTLPATTVFRLDREERHGDISTPLGNLSKLMESTDPSKRLRELIIDFR 600
DNA2_HUMAN      MTTVTCLLDRNLSVLPESTLFRLDQEEKNCDIDTPLGNLSKLMENTFVSKKLRDLIIDFR 599
                 ::**********.** :*:****:**:: **.***********.*  **:**:******

DNA2_MOUSE      EPQFIAYLSSVLPHDAKDTVANILKGLNKPQRQAMKRVLLSKDYTLIVGMPGTGKTTTIC 660
DNA2_HUMAN      EPQFISYLSSVLPHDAKDTVACILKGLNKPQRQAMKKVLLSKDYTLIVGMPGTGKTTTIC 659
                *****:*************** **************:***********************

DNA2_MOUSE      ALVRILSACGFSVLLTSYTHSAVDNILLKLAKFKVGFLRLGQSHKVHPDIQKFTEEEICR 720
DNA2_HUMAN      TLVRILYACGFSVLLTSYTHSAVDNILLKLAKFKIGFLRLGQIQKVHPAIQQFTEQEICR 719
                :***** ***************************:******* :**** **:***:****

DNA2_MOUSE      SRSIASLAHLEELYNSHPIVATTCMGINHPIFSRKTFDFCIVDEASQISQPVCLGPLFFS 780
DNA2_HUMAN      SKSIKSLALLEELYNSQLIVATTCMGINHPIFSRKIFDFCIVDEASQISQPICLGPLFFS 779
                *:** *** *******: ***************** ***************:********

DNA2_MOUSE      RRFVLVGDHQQLPPLVVNREARALGMSESLFKRLERNESAVVQLTVQYRMNRKIMSLSNK 840
DNA2_HUMAN      RRFVLVGDHQQLPPLVLNREARALGMSESLFKRLEQNKSAVVQLTVQYRMNSKIMSLSNK 839
                ****************:******************:*:************* ********

DNA2_MOUSE      LTYAGKLECGSDRVANAVLALPNLKDARLSLQLYADYSDSPWLAGVLEPDNPVCFLNTDK 900
DNA2_HUMAN      LTYEGKLECGSDKVANAVINLRHFKDVKLELEFYADYSDNPWLMGVFEPNNPVCFLNTDK 899
                *** ********:*****: * ::**.:*.*::******.*** **:**:**********

DNA2_MOUSE      VPAPEQVENGGVSNVTEARLIVFLTSTFIKAGCSPSDIGVIAPYRQQLRIISDLLARSSV 960
DNA2_HUMAN      VPAPEQVEKGGVSNVTEAKLIVFLTSIFVKAGCSPSDIGIIAPYRQQLKIINDLLAR-SI 958
                ********:*********:******* *:**********:********:**.***** *:

DNA2_MOUSE      GMVEVNTVDKYQGRDKSLILVSFVRSNEDGTLGELLKDWRRLNVALTRAKHKLILLGSVS 1020
DNA2_HUMAN      GMVEVNTVDKYQGRDKSIVLVSFVRSNKDGTVGELLKDWRRLNVAITRAKHKLILLGCVP 1018
                *****************::********:***:*************:***********.*.

DNA2_MOUSE      SLKRFPPLGTLFDHLNAEQLILDLPSREHESLSHILGDCQRD 1062
DNA2_HUMAN      SLNCYPPLEKLLNHLNSEKLIIDLPSREHESLCHILGDFQRE 1060
                **: :*** .*::***:*:**:**********.***** **:
```

Figure S1. Sequence alignment of the full-length mouse and human DNA2 proteins. Amino acid identities are indicated by asterisks below the sequences. The P-loop region (red) is flanked by a preceding a β-strand (green) and a following α-helix (blue). T652 (highlighted in yellow) is the residue investigated in this study.

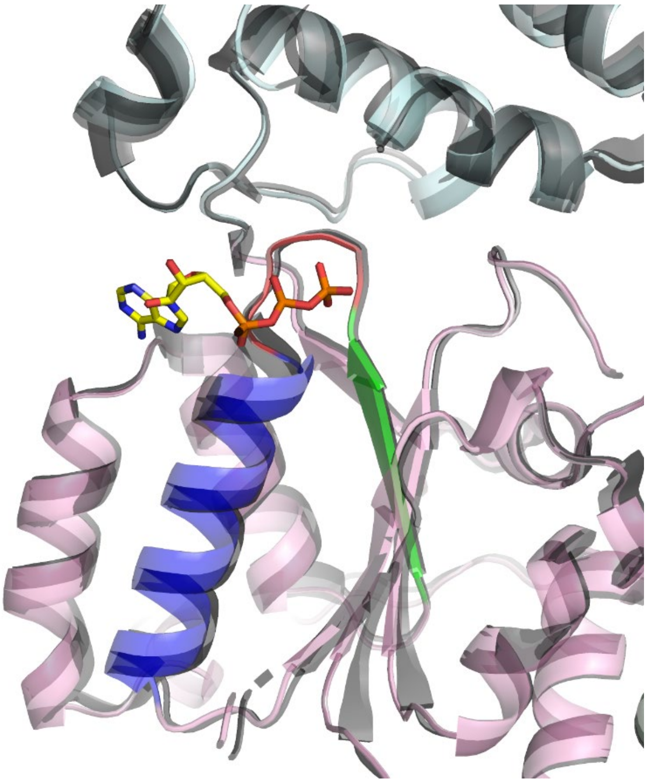

```
MOUSE    631 QRQAMKRVLLSKDYTLIVGMPGTGKTTICALVRILSACG 670
HUMAN    630 QRQAMKKVLLSKDYTLIVGMPGTGKTTICTLVRILYACG 669
             ******:**********************:********
```

Figure S2. Superposition of the ATP/ADP-binding site of mouse and human DNA2 structures. The P-loop region (red) is flanked by a preceding a β-strand (green) and a following α-helix (blue). The remaining regions of the human DNA2 structure are shown in magenta (helicase domain 1A) and cyan (helicase domain 2A), and the corresponding regions of the mouse DNA2 structure are shown in gray. T652 (highlighted in yellow) is the residue investigated in this study.

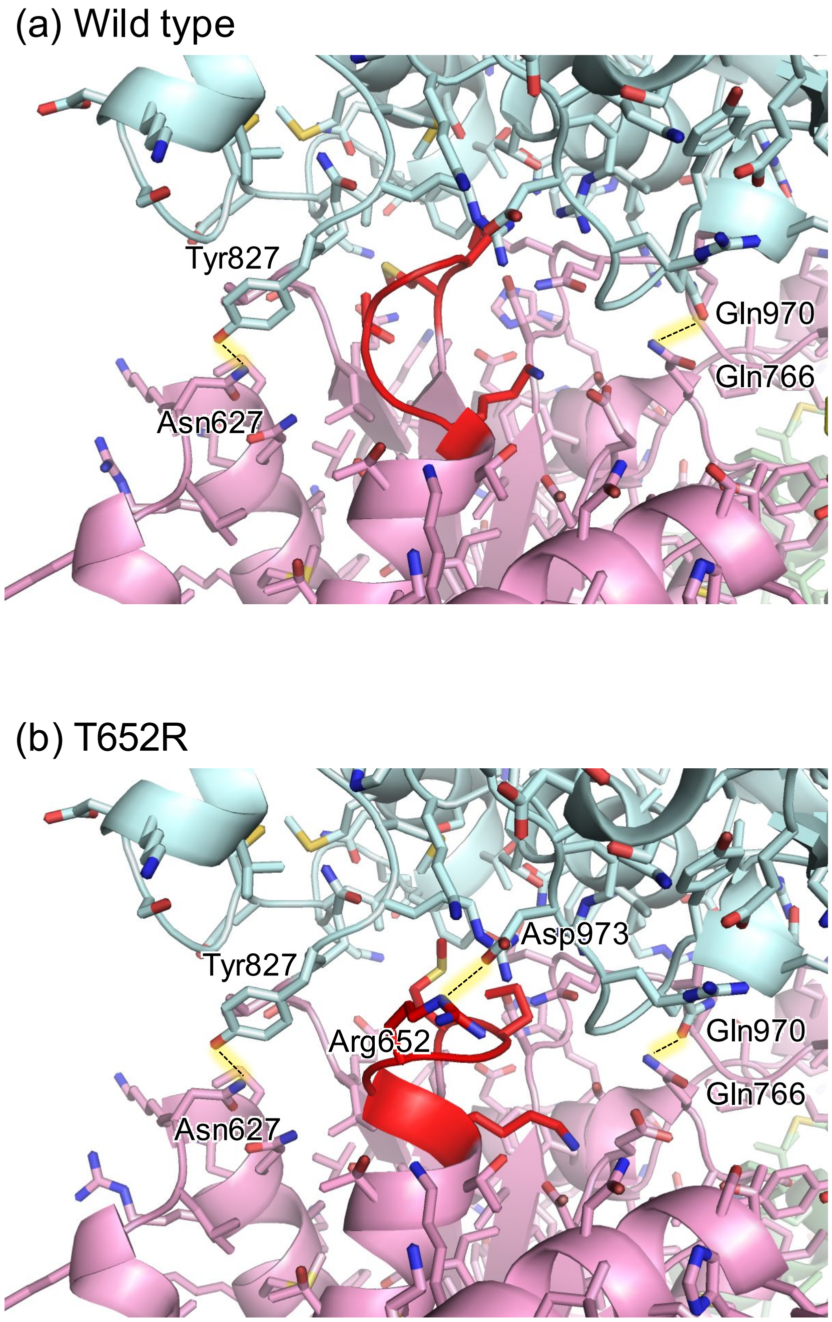

Figure S3. Structural interactions stabilizing the closed conformation of DNA2. (a) Wild-type human DNA2 structure. (b) T652R mutant DNA2 structure. In the mutant, the introduced Arg652 residue (red) forms a salt bridge with Asp973, linking helicase domains 1A (magenta) and 2A (cyan) and stabilizing the closed conformation. This additional inter-domain interaction, absent in the wild type, likely restricts domain motion required for reopening after ATP hydrolysis. H-bonds are shown as black dotted lines highlighted in yellow.
